# Progressive Small Vessel Disease Burden as a Diagnostic of Central Nervous System-Restricted Microscopic Polyangiitis: A Case Report and Review of the Literature

**DOI:** 10.7759/cureus.30462

**Published:** 2022-10-19

**Authors:** Haruka Hoshi, Hajime Ikenouchi, Naoki Yamamoto, Tatsuo Miyamoto, Kaoru Endo

**Affiliations:** 1 Neurology, Sendai City Hospital, Sendai, JPN

**Keywords:** inflammatory reaction, microscopic polyangiitis, vasculitis, cerebral microbleeds, small vessel disease

## Abstract

Microscopic polyangiitis (MPA) is a type of anti-neutrophil cytoplasmic antibody (ANCA)-associated vasculitis linked to myeloperoxidase (MPO), usually accompanied by pulmonary and renal lesions. MPA sometimes causes central nervous system (CNS) involvement such as cerebral infarction. Herein, we report a case of a 72-year-old man with a headache. He had an unknown cause of the elevated inflammatory response. Magnetic resonance imaging (MRI) showed multiple cerebral infarctions in the small vessel region in the right basal ganglia with multiple cerebral microbleeds (cMBs). After admission, his left hemiparesis and consciousness disturbance gradually deteriorated. A follow-up MRI on day 18 showed increased multiple cerebral infarctions in small vessel regions with increased cMBs. Additional blood tests revealed positive MPO-ANCA. Although there were no findings suggestive of active renal or pulmonary involvement or peripheral neuropathy, we diagnosed him as having MPA-associated CNS-restricted vasculitis. CNS involvement of MPA is relatively rare but is associated with a high small vessel disease (SVD) burden. In addition to the unknown cause of inflammatory response, the multiple cMBs increase and a short-term recurrence of cerebral infarctions in the bilateral thalamus and basal ganglia was the clue for the diagnosis of CNS-restricted vasculitis. It is difficult to diagnose MPA vasculitis when lesions are restricted to the CNS. In the absence of lesions other than SVD, MPA-associated CNS vasculitis should be suspected in patients with progressive SVD burden and elevated inflammatory response.

## Introduction

Central nervous system (CNS) vasculitis sometimes occurs as a part of systemic vasculitis. Anti-neutrophil cytoplasmic antibody (ANCA)-associated vasculitis is characterized by pathogenic ANCA production and causes systemic small vessel vasculitis [1]. Microscopic polyangiitis (MPA) is a type of ANCA-associated vasculitis linked to myeloperoxidase (MPO), usually accompanied by pulmonary and renal lesions [1]. In cases of MPA, CNS involvement may sometimes manifest itself as cerebral infarction in the small vessel region [2]. However, CNS-restricted MPA was extremely rare, and no report shows progressive small vessel infarction and cerebral microbleeds as initial findings of CNS-restricted MPA. Here, we report a case of CNS-restricted MPA whose diagnosis was prompted by a short period of progressive small vessel disease (SVD).

## Case presentation

**Figure 1:**
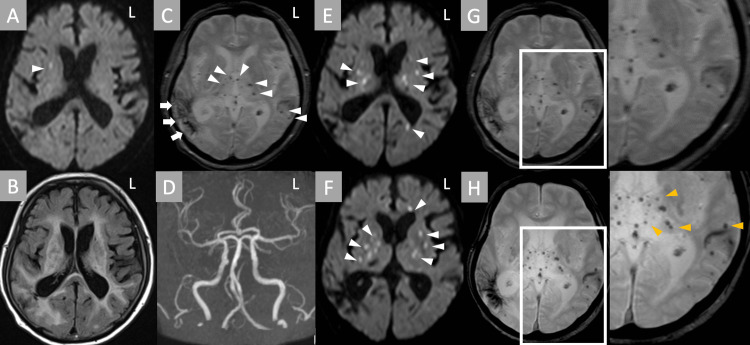
Imaging results. A: Axial diffusion-weighted imaging (DWI) showed hyperintense lesions in the right basal ganglia (arrowhead). B: Fluid attenuated inversion recovery showed broad white matter lesions and severe subcortical white matter lesions. C: T2*-weighted imaging (T2*WI) showed multiple cerebral microbleeds both in the thalamus and subcortical region (arrowheads), in addition to the pre-existing intracerebral hemorrhage (arrows). D: MRA showed no occlusion or stenosis in the major cerebral arteries. E, F: Axial DWI showed multiple hyperintense lesions in the bilateral small vessel territory (arrowheads). G, H: Compared with initial T2*WI (G), cerebral microbleeds became more pronounced in the follow-up MRI (H) (arrowheads). L, left; MRA, magnetic resonance angiography.

A 72-year-old man was brought to our hospital because of an acute headache. He had a history of hypertension and subcortical intracerebral hemorrhage in the right hemisphere, dating back to a year before admission. Although left hemispatial neglect remained, he was independent in daily living. He had taken antihypertensive agents. At the presentation, the physical examination was normal. On neurological examination, in addition to left hemispatial neglect, he had a mild headache and disorientation. He had no dysarthria, hemiparesis, or sensory disturbance. His deep tendon reflex was normal. Although he had no fever or suggestive findings of infection, blood tests revealed elevated white blood cell counts (9,900/μl) and elevated C-reactive protein (10.14 mg/dl). Blood tests did not show renal dysfunction, and whole-body computed tomography did not detect any lesions. Brain magnetic resonance imaging (MRI) exposed a small cerebral infarction in the right basal ganglia (Figure 1A) with broad white matter change (Figure 1B). He also had multiple cerebral microbleeds (cMBs) in bilateral basal ganglia, thalamus, and subcortical regions, in addition to the pre-existing subcortical hemorrhage in the right temporal lobe (Figure 1C). Magnetic resonance angiography showed no stenosis or occlusions in major arteries (Figure 1D). After several days, his inflammatory response did not improve, while left hemiparesis and drowsiness gradually progressed. A follow-up brain MRI on day 18 showed increased multiple cerebral infarctions restricted in the bilateral thalamus and basal ganglia (Figures 1E, 1F). Deep and lobar cMBs also increased (Figures 1G, 1H). Cerebrospinal fluid analysis revealed slight pleocytosis (7/μl) with slightly elevated protein concentration (49 mg/dl). Additional blood tests revealed positive MPO-ANCA (77 IU/dl). Blood tests, urine tests, and body computed tomography did not reveal any active renal or pulmonary lesions. There were also no suggestive findings of peripheral neuropathy. Based on the findings of positive MPO-ANCA, unknown cause of systemic inflammation, multiple infarcts restricted to the bilateral thalamus and basal ganglia with multiple cMBs, and abnormal CSF findings, we diagnosed him as having MPA-associated CNS-restricted vasculitis. Intravenous and oral corticosteroid therapy and cyclophosphamide gradually improved consciousness disturbance, elevated inflammatory response, and MPO-ANCA levels. On day 120, he was transferred to the hospital for rehabilitation due to left hemiplegia and cognitive dysfunction.

## Discussion

We describe a case of CNS-restricted MPA as the initial findings of cerebral infarction restricted in the small vessel region. This diagnosis was prompted by an unknown cause of elevated inflammatory response and progressive multiple small vessel infarctions with multiple cMBs in very short periods. MPA is a small vessel vasculitis caused by MPO-ANCA. MPA usually presents with renal and pulmonary lesions [1]. CNS involvement as a neurological manifestation was reported in only 17%, such as patchy meningitis, parenchymal mass lesions, posterior reversible encephalopathy syndrome, cerebral infarction, and intracerebral hemorrhage [2,3]. Reflecting its aspect of small vessel vasculitis, stroke in MPA is accompanied by severe white matter change, small vessel infarctions, and multiple deep and lobar cMBs [3-6], suggesting a higher SVD burden [7]. Therefore, a higher SVD burden could occur and progress in MPA as its aspects of chronic and continuous inflammation of small vessels [2,4]. In the present case, all multiple infarctions at recurrence were restricted in the small vessel region. This imaging finding suggests small vessel vasculitis, rather than embolic stroke. In addition, multiple cMBs are usually seen in hypertensive changes or cerebral amyloid angiopathy [8]. However, in the present case, multiple cMBs increased parallel with the recurrence of cerebral infarction in very short periods. Although we could not conduct the biopsy for the confirmation of vasculitis, this SVD progression in a short period would be abnormal in hypertensive change or amyloid angiopathy, suggesting that progressive multiple infarctions and cMBs would reflect CNS vasculitis [4].

Cerebral infarction as an isolated finding of MPA is very rare. There have been four cases of CNS-restricted MPA with cerebral infarction in the current study and the literature (Table 1) [9-11]. In all cases, cerebral infarction was predominantly located in the small vessel region such as the thalamus, basal ganglia, and corona radiata. An elevated inflammatory response was observed in all cases. The renal or pulmonary lesion was not observed in all cases. In two cases, the confirmation of vasculitis was made by the sural nerve or skin biopsy [9,10]. In two cases including our case, disease remission and decrement of ANCA level by immunosuppression therapy led to the diagnosis [11]. Three cases including our case showed a recurrence of cerebral infarction or intracerebral hemorrhage [9,11]. Except for one case who died of intracerebral hemorrhage [9], disease remission was achieved by immunosuppression therapy. Other than our case, there were no cases of progressive SVD burden such as increased cerebral infarction and cMBs in short periods.

**Table 1:** The previous cases of CNS-restricted MPA ANCA, anti-neutrophil cytoplasmic antibody; CI, cerebral infarction; cMBs, cerebral microbleeds; IVCY, intravenous cyclophosphamide; mPSL, methylprednisolone; CNS, central nervous system; MPA, microscopic polyangiitis; ICH, intracerebral hemorrhage.

Previous cases	Age	Sex	Initial lesion type	Lesion site	Elevated inflammatory response	Other lesions	Diagnosis of vasculitis	Progression or recurrence of CNS lesion	Recurrence type	Treatment	Outcome
Ito et al., 2006 [9]	56	Male	CI	Corona radiata	Yes	No	Sural nerve biopsy	Yes	ICH	mPSL	Death
Tang et al., 2009 [10]	55	Male	CI	Basal ganglia	Yes	Skin rash	Skin biopsy	No	--	mPSL IVCY	Remission
Wakisaka et al., 2014 [11]	73	Male	CI	Basal ganglia, corona radiata, and subcortical	Yes	No	Positive ANCA treatment response	Yes (10 days after admission)	CI	mPSL	Remission
The present case	72	Male	CI	Thalamus and basal ganglia	Yes	No	Positive ANCA treatment response	Yes (18 days after admission)	CI and increased cMBs	mPSL IVCY	Remission

The mechanism of CNS-restricted MPA has not been fully understood. One possibility is that cerebral infarction is only the initial symptom, and other organ lesions could appear with follow-up. It is difficult to diagnose MPA when lesions are restricted to the CNS. The delay in the diagnosis of vasculitis would result in negative health consequences [12]. In addition, intracerebral hemorrhage in MPA sometimes can be critical and fatal [9,13]. Therefore, early diagnosis and treatment should be desired. Our case would suggest that CNS vasculitis should be considered in patients with unknown causes of elevated inflammatory response alongside higher and progressive SVD burden.

## Conclusions

CNS-restricted MPA is a rare condition and sometimes difficult to diagnose. In the present case, the unknown cause of inflammatory response and progressive small vessel infarctions and cMBs in the short period was the clue for the diagnosis. Intracerebral hemorrhage in MPA sometimes can be critical and fatal. Even though there was no lesion other than SVD, clinicians should be aware of MPA-associated CNS vasculitis, especially in the high and progressive SVD burden and unknown cause of the inflammatory response.
